# A systematic review and meta-analysis: Does hepatitis C virus infection predispose to the development of chronic kidney disease?

**DOI:** 10.18632/oncotarget.12896

**Published:** 2016-10-25

**Authors:** Min Li, Peiyuan Wang, Chunhua Yang, Wenguo Jiang, Xiaodan Wei, Xinbo Mu, Xuri Li, Jia Mi, Geng Tian

**Affiliations:** ^1^ Medicine and Pharmacy Research Center, Binzhou Medical University, Yantai, Shandong, China; ^2^ Institute of Imaging, Yantai Affiliated Hospital of Binzhou Medical University, Yantai, Shandong, China; ^3^ Personnel Department, Binzhou Medical University, Yantai, Shandong, China

**Keywords:** hepatitis C virus, chronic kidney disease, meta-analysis, effect estimate

## Abstract

We aimed to meta-analytically assess the predisposition of hepatitis C virus (HCV) infection to the occurrence and severity of chronic kidney disease (CKD). Two authors independently searched articles and abstracted information. Odds ratio (OR) or hazard ratio (HR) along with 95% confidence interval (CI) was converged separately in 12 longitudinal (1,972,044 subjects) and 15 cross-sectional (937,607 subjects) studies. Overall effect estimate was remarkably significant in longitudinal studies (HR, 95% CI, *P:* 1.45, 1.23-1.71, < 0.001), in contrast to that in cross-sectional studies (OR, 95% CI, *P:* 1.25, 0.90-1.73, 0.188), with obvious heterogeneity (*I*^2^ > 95%). HCV infection was also associated with an 1.54-fold (95% CI, *P:* 1.27-1.87, < 0.001) increased risk of having prevalent proteinuria. In longitudinal studies with estimated glomerular filtration rate (eGFR) < 60, < 30 and < 15 ml/min/1.73m^2^, the corresponding HR was 1.39 (95% CI, *P:* 1.14-1.69, 0.001), 1.79 (0.91-3.51, 0.091) and 2.30 (1.26-4.19, 0.007). Further grouping the longitudinal studies by median follow-up time at 5 years revealed that the effect estimate was reinforced in long-term studies (HR, 95% CI, *P:* 1.86, 1.19-2.89, 0.006; *I*^2^=98.1%) relative to that in short-term studies (1.21, 1.03-1.43, 0.024; 92.0%). In conclusion, our findings demonstrate the significant risk of experiencing incident CKD after HCV infection, with the lower eGFR and longer HCV exposure time entailing a greater risk.

## INTRODUCTION

Hepatitis C virus (HCV) is a blood-borne virus and its infection imposes a global health burden in both developed and developing countries [1]. HCV is a major cause of advanced liver-related outcomes such as cirrhosis, hepatocellular carcinoma and a growing spectrum of extra-hepatic complications such as dermatological, rheumatological and haematological disorders, as well as kidney insufficiency [2, 3]. It is estimated that nearly half of HCV-seropositive patients are diagnosed to experience at least one extra-hepatic complication [4]. There is competing evidence that HCV has the feasibility of entry and replication in kidney tissue, ending up with many severe endpoints such as acute interstitial nephritis and focal segmental sclerosis [5]. Echoing from epidemiological observations, there was a close relationship between HCV infection and chronic kidney disease (CKD), while current literature is teeming with inconsistent results, with positive association being reported in some [6, 7] but not all [8, 9] studies. CKD was defined as kidney damage (the presence of albuminuria) or decreased estimated glomerular filtration rate (eGFR < 60 ml/min/1.73 m^2^). A previous meta-analysis by Fabrizi et al summarized the predisposition of HCV-infected patients to CKD and they failed to produce any observable significance [10]. More recently, Park et al [11] and Fabrizi et al [12] have separately conducted an updated meta-analysis of this project and demonstrated an increased risk for the development of CKD in HCV-infected patients compared to uninfected individuals. In view of these inconclusive findings, a comprehensive assessment is urgently required and we hence hypothesized that HCV infection was a significant risk factor for the development of CKD. To explore this hypothesis further, after *de novo* evaluation, we identified additional five articles in English-language literature that were not incorporated in previous meta-analyses [10–12]. In addition, considering the intractable confounding or recall bias inherited in cross-sectional studies, we determined to meta-analytically assess the predisposition of HCV infection to the occurrence and severity of CKD separately in cross-sectional and longitudinal studies, and further seek other possible interpretations for the obvious heterogeneity obsessing existing meta-analyses.

## RESULTS

### Eligible studies and characteristics

The selection process of all eligible studies is shown in Supporting Figure S1. A total of 545 articles were identified after searching four electronic databases with predefined key terms, and 22 eligible articles involving 12 longitudinal studies (1,972,044 subjects) and 15 cross-sectional studies (937,607 subjects) were finally analyzed [6–9, 13–30]. After treating the studies with different eGFR cutoffs (eGFR < 60, < 30, < 15 ml/min/1.73m^2^) and/or proteinuria individually, there were 38 studies (5,077,110 subjects, 15 longitudinal studies and 23 cross-sectional studies) in the corresponding subgroup analysis and their study characteristics are presented in Table 1 and Table 2.

**Figure 1:**
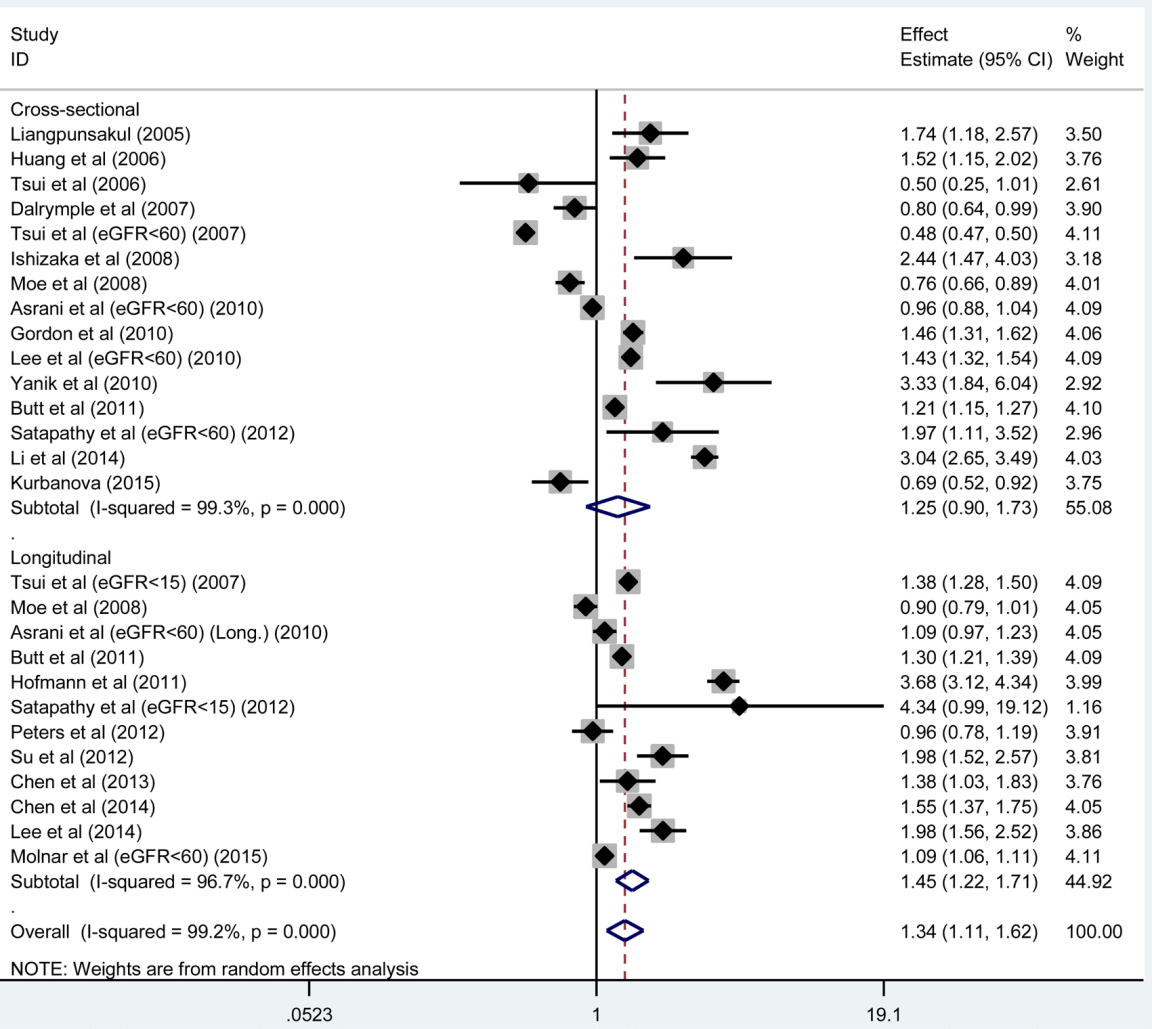
The forest plot for the prevalence and incidence of CKD conferred by the presence of HCV infection The effect estimate (odds ratio [OR]) is presented by the vertical central axis of the solid diamond for each study, and its 95% confidence interval (CI) is denoted by the left and right extremes of the horizontal central line through the solid diamond. The vertical broken axis of the hollow diamond represents the overall effect estimate. The solid vertical line is set at the null effect estimate (OR = 1.0). The left and right of x-axis represent the decreased and increased CKD risk, respectively.

**Table 1:** The baseline characteristics of all qualified studies enrolled in this meta-analysis

Author (Index) (year)	Country	Collection time	Cohort sample size	Included sample size	Definition	Median follow-up (years)	Unexposed		Exposed	
							Total	Events	Total	Events
**Cross-sectional studies**										
Liangpunsakul (2005)	US	1988-1994	15,336	1,357	Proteinuria	0	995	75	362	45
Huang et al (2006)	Taiwan	2002-2004	10,975	8,571	Proteinuria	0	8,004	557	567	58
Tsui et al (2006)	US	1988-1994	34,000	15,029	eGFR<60 ml/min/1.73m2	0	14,663	631	366	8
Tsui et al (Proteinuria) (2006)	US	1988-1994	34,000	15,029	Proteinuria	0	14,663	1,760	366	55
Dalrymple et al (2007)	US	1999-2004	39,574	25,782	eGFR<60 ml/min/1.73m2	0	23,854	1,423	1,928	93
Tsui et al (eGFR<60) (C) (2007)	US	2000-2001	2,352,584	474,369	eGFR<60 ml/min/1.73m2	0	421,495	71,654	52,874	4,759
Tsui et al (eGFR<30) (C) (2007)	US	2000-2001	2,352,584	474,369	eGFR<30 ml/min/1.73m2	0	421,495	8,430	52,874	1,058
Ishizaka et al (2008)	Japan	2004-2006	12,535	12,405	eGFR<60 ml/min/1.73m2	0	12,333	1,887	72	22
Ishizaka et al (Proteinuria) (2008)	Japan	2004-2006	12,535	12,405	Proteinuria	0	12,333	1,157	72	14
Moe et al (C.-C.) (2008)	US	1994-2004	19,303	13,139	eGFR<60 ml/min/1.73m2	0	9,201	745	3,938	248
Asrani et al (eGFR<60) (C) (2010)	US	2003-2006	6,000,000	167,569	eGFR<60 ml/min/1.73m2	0	154,185	8,172	13,384	682
Asrani et al (eGFR<30) (C) (2010)	US	2003-2006	6,000,000	167,569	eGFR<30 ml/min/1.73m2	0	154,185	370	13,384	29
Asrani et al (eGFR<15) (C) (2010)	US	2003-2006	6,000,000	167,569	eGFR<15 ml/min/1.73m2	0	154,185	123	13,384	13
Gordon et al (2010)	US	1997-2006	79,492	67,063	eGFR<60 ml/min/1.73m2	0	64,006	6,666	3,057	443
Lee et al (eGFR<60) (2010)	Taiwan	2004	55,780	49,048	eGFR<60 ml/min/1.73m2	0	43,859	6,249	5,189	994
Lee et al (eGFR<30) (2010)	Taiwan	2004	55,780	49,048	eGFR<30 ml/min/1.73m2	0	43,859	333	5,189	56
Lee et al (Proteinuria) (2010)	Taiwan	2004	55,780	49,048	Proteinuria	0	43,859	2,385	5,189	332
Yanik et al (2010)	US	1998-2008	4,376	900	Proteinuria	0	129	13	772	210
Butt et al (C.-C.) (2011)	US	2001-2006	68,285	43,139	eGFR<60 ml/min/1.73m2	0	25,137	3,738	18,002	3,140
Satapathy et al (eGFR<60) (2012)	US	2003-2006	2,415	865	eGFR<60 ml/min/1.73m2	0	313	16	552	53
Li et al (2014)	Taiwan	2010-2011	24,642	24,642	eGFR<60 ml/min/1.73m2	0	22,943	1,398	1,699	280
Kurbanova (2015)	US	1999-2012	33,729	33,729	eGFR<60 ml/min/1.73m2	0	33,070	3,523	659	50
Kurbanova (Proteinuria) (2015)	US	1999-2012	33,729	33,729	Proteinuria	0	33,070	4,183	659	112
**Longitudinal studies**										
Tsui et al (eGFR<15) (L.) (2007)	US	2000-2001	2,352,584	474,369	eGFR<15 ml/min/1.73m2	3.4	421,495	4,393	52,874	760
Moe et al (Long.) (2008)	US	1994-2004	19,303	7,038	eGFR<60 ml/min/1.73m2	3.5	4,795	NR	2,243	NR
Asrani et al (eGFR<60) (L) (2010)	US	2003-2006	6,000,000	88,822	eGFR<60 ml/min/1.73m2	2.1	80,759	2,826	8,063	306
Asrani et al (eGFR<30) (L) (2010)	US	2003-2006	6,000,000	88,822	eGFR<30 ml/min/1.73m2	2.1	80,759	56	8,063	10
Asrani et al (eGFR<15) (L) (2010)	US	2003-2006	6,000,000	88,822	eGFR<15 ml/min/1.73m2	2.1	80,759	8	8,063	2
Butt et al (Long.) (2011)	US	2001-2006	68,285	43,139	eGFR<60 ml/min/1.73m2	3.15	25,137	NR	18,002	NR
Hofmann et al (2011)	Europe	1990-2006	258,000	223,536	eGFR<60 ml/min/1.73m2	9.3	198,124	443	25,412	208
Satapathy et al (eGFR<15) (2012)	US	2003-2006	2,415	865	eGFR<15 ml/min/1.73m2	7	313	2	552	15
Peters et al (2012)	Europe	1994-2011	16,594	8,235	eGFR<60 ml/min/1.73m2	4.39	6,183	375	2,052	120
Su et al (2012)	Taiwan	2000-2005	1,000,000	37,746	eGFR<15 ml/min/1.73m2	5.58	31,455	196	6,291	77
Chen et al (2013)	Taiwan	1998-2004	1,000,000	15,910	eGFR<60 ml/min/1.73m2	5.92	12,728	187	3,182	64
Chen et al (2014)	Taiwan	1996-2010	1,000,000	47,150	eGFR<60 ml/min/1.73m2	7.43	37,720	960	9,430	367
Lee et al (2014)	Taiwan	2002-2009	4,321	4,185	eGFR<60 ml/min/1.73m2	2.2	3,868	891	317	118
Molnar et al (eGFR<60) (2015)	US	2004-2006	4,444,699	1,021,049	eGFR<60 ml/min/1.73m2	8	920,531	95,837	100,518	11,271
Molnar et al (eGFR<15) (2015)	US	2005-2006	4,444,699	1,021,049	eGFR<15 ml/min/1.73m2	8	920,531	2,479	100,518	904

Note. (C): cross-sectional studies; (L): longitudinal studies; CKD, chronic kidney disease; eGFR: estimated glomerular filtration rate; NR: not reported.

**Table 2:** The baseline characteristics of all study populations included in this meta-analysis

Author (Index) (year)	EE*; 95% CI	Adj-EE*; 95% CI	Age (years)		Male (%)		DM		Hypertension	
			Unexpo.	Expo.	Unexpo.	Expo.	Unexpo.	Expo.	Unexpo.	Expo.
**Cross-sectional studies**										
Liangpunsakul (2005)	1.51; 1.67-2.15	1.99; 1.38-2.85	43	43	61	62	4.7	10.5	25	32
Huang et al (2006)	1.52; 1.15-2.03	NR	55.2	55.2	43.2	43.2	12.5	12.5	33.4	33.4
Tsui et al (2006)	0.45; 0.24-0.85	0.89; 0.49-1.62	NR	NR	47.00	67.00	5.00	5.00	31.00	27.00
Tsui et al (Proteinuria) (2006)	1.29; 0.86-1.93	1.38; 0.91-2.07	NR	NR	47.00	67.00	5.00	5.00	31.00	27.00
Dalrymple et al (2007)	1.08; 0.88-1.33	1.08; 0.88-1.33	58.00	53.00	91.00	96.00	22.00	19.00	69.00	61.00
Tsui et al (eGFR<60) (C) (2007)	0.48; 0.47-0.50	NR	59.00	52.00	94.00	97.00	26.00	21.00	59.00	47.00
Tsui et al (eGFR<30) (C) (2007)	1.00; 0.94-1.07	NR	59.00	52.00	94.00	97.00	26.00	21.00	59.00	47.00
Ishizaka et al (2008)	2.46; 1.54-3.94	1.83; 1.10-3.05	53.1	59.2	64.19	62.5	NR	NR	NR	NR
Ishizaka et al (Proteinuria) (2008)	2.33; 1.30-4.19	2.00; 1.06-3.76	53.1	59.2	64.19	62.5	NR	NR	NR	NR
Moe et al (C.-C.) (2008)	0.76; 0.66-0.89	0.69; 0.62-0.77	41.20	43.60	44.10	60.40	22.60	23.40	46.50	50.80
Asrani et al (eGFR<60) (C) (2010)	0.92; 0.79-1.08	0.92; 0.79-1.08	40.40	47.80	43.80	60.10	6.70	9.60	7.60	9.70
Asrani et al (eGFR<30) (C) (2010)	0.90; 0.62-1.32	NR	40.40	47.80	43.80	60.10	6.70	9.60	7.60	9.70
Asrani et al (eGFR<15) (C) (2010)	1.22; 0.69-2.16	NR	40.40	47.80	43.80	60.10	6.70	9.60	7.60	9.70
Gordon et al (2010)	1.46; 1.31-1.62	NR	48.00	52.00	48.20	62.30	NR	NR	NR	NR
Lee et al (eGFR<60) (2010)	1.36; 1.27-1.46	1.26; 1.17-1.38	60.80	64.30	31.00	29.30	9.70	10.50	31.00	32.70
Lee et al (eGFR<30) (2010)	1.43; 1.07-1.90	NR	60.80	64.30	31.00	29.30	9.70	10.50	31.00	32.70
Lee et al (Proteinuria) (2010)	1.19; 1.06-1.34	1.14; 1.00-1.3	60.80	64.30	31.00	29.30	9.70	10.50	31.00	32.70
Yanik et al (2010)	2.07; 1.59-4.58	1.84; 1.03-3.27	48.9	48.9	65.4	65.4	10.8	10.8	38.7	38.7
Butt et al (C.-C.) (2011)	1.21; 1.15-1.27	NR	52.80	51.90	97.30	97.30	26.60	22.90	60.80	52.40
Satapathy et al (eGFR<60) (2012)	1.97; 1.11-3.51	NR	50.00	50.00	64.20	68.30	16.30	19.00	37.40	39.30
Li et al (2014)	1.24; 1.05-1.48	1.24; 1.05-1.48	41.70	42.40	52.80	42.40	NR	NR	NR	NR
Kurbanova (2015)	0.69; 0.47-1.02	0.88; 0.57-1.37	49.5	50.8	48	63.9	12.2	14.4	36.4	43.7
Kurbanova (Proteinuria) (2015)	1.40; 1.08-1.81	1.50; 1.08-2.08	49.5	50.8	48	63.9	12.2	14.4	36.4	43.7
**Longitudinal studies**										
Tsui et al (eGFR<15) (L.) (2007)	1.39; 1.28-1.50	1.68; 1.54-1.82	59.00	52.00	94.00	97.00	26.00	21.00	59.00	47.00
Moe et al (Long.) (2008)	0.90; 0.79-1.02	0.90; 0.79-1.02	41.30	44.10	45.40	58.20	18.20	19.90	41.70	44.70
Asrani et al (eGFR<60) (L) (2010)	1.09; 0.97-1.23	NR	43.20	48.70	40.80	59.20	10.30	12.40	11.10	12.30
Asrani et al (eGFR<30) (L) (2010)	1.79; 0.91-3.51	NR	43.20	48.70	40.80	59.20	10.30	12.40	11.10	12.30
Asrani et al (eGFR<15) (L) (2010)	2.50; 0.53-11.8	NR	43.20	48.70	40.80	59.20	10.30	12.40	11.10	12.30
Butt et al (Long.) (2011)	1.30; 1.23-1.37	1.30; 1.23-1.37	52.80	51.90	97.30	97.30	26.60	22.90	60.80	52.40
Hofmann et al (2011)	3.68; 3.12-4.34	NR	NR	37.60	69.10	69.10	NR	3.70	NR	NR
Satapathy et al (eGFR<15) (2012)	4.34; 0.99-19.12	NR	50.00	50.00	64.20	68.30	16.30	19.00	37.40	39.30
Peters et al (2012)	0.96; 0.78-1.19	NR	42.00	39.00	75.90	67.80	4.80	3.70	25.90	14.70
Su et al (2012)	1.53; 1.17-2.01	1.53; 1.17-2.01	NR	NR	50.50	50.50	NR	NR	NR	NR
Chen et al (2013)	1.75; 1.27-2.43	1.75; 1.27-2.43	NR	NR	50.90	50.90	7.70	0.00	13.60	0.00
Chen et al (2014)	1.28; 1.12-1.46	1.28; 1.12-1.46	NR	NR	49.60	49.60	14.40	25.20	28.60	34.10
Lee et al (2014)	1.32; 1.07-1.62	1.32; 1.07-1.62	61.77	64.53	59.40	47.60	35.60	43.50	11.10	9.20
Molnar et al (eGFR<60) (2015)	1.15; 1.12-1.17	1.15; 1.12-1.17	55.00	53.00	92.00	96.00	21.00	21.00	54.00	53.00
Molnar et al (eGFR<15) (2015)	1.98; 1.81-2.16	1.98; 1.81-2.16	55.00	53.00	92.00	96.00	21.00	21.00	54.00	53.00

Note. (C): cross-sectional studies; (L): longitudinal studies; Unexpo.: unexposed; Expo.: exposed; eGFR: estimated glomerular filtration rate; EE: effect estimate; Adj-EE: adjusted effect estimate; 95% CI: 95% confidence interval; NR: not reported. *EE refers to odds ratio in cross-sectional studies and hazard ratio in longitudinal studies.

The number of studies testing the prevalence or incidence of proteinuria, eGFR < 60, < 30 and < 15 ml/min/1.73m^2^ was 7, 21, 4 and 6, respectively. Twenty-five of 38 studies were conducted in the U.S., 9 in Taiwan, 2 in Japan and 2 in European countries. Adjusted effect estimate and its 95% CI were reported in 23 studies. In 15 longitudinal studies, the median follow-up period ranged from 2.1 years [9] to 9.3 years [8].

### Effect estimates

Considering the methodological distinction between cross-sectional and longitudinal studies, we analyzed them separately in this study. To avoid repeated incorporation, only study with eGFR < 60 ml/min/1.73m^2^ was retained in case of different eGFR cutoffs recorded in the same article, and there were 12 and 15 unduplicated studies with longitudinal and cross-sectional designs, respectively. Overall effect estimate was remarkably significant in longitudinal studies (HR, 95% CI, *P* : 1.45, 1.23-1.71, < 0.001), in contrast to that in cross-sectional studies (OR, 95% CI, *P* : 1.25, 0.90-1.73, 0.188), while there was strong evidence of heterogeneity (both *I*^2^ > 95%) (Figure 1). The power to identify the significant association in longitudinal studies was over 99.9%. After restricting analysis to the studies with adjusted effect estimates, the magnitude of risk was weakened, but significance was still persisted in 8 longitudinal studies (HR, 95% CI, *P* : 1.31, 1.15-1.48, < 0.001), relative to that in 10 cross-sectional studies (OR, 95% CI, *P* : 1.15, 0.93-1.43, 0.197), with obvious heterogeneity (both *I*^2^ > 90%).

To investigate the impact of specific clinical differences between studies, we performed a set of stratified analyses according to CKD subtype, country and median follow-up period (for longitudinal studies only), respectively (Table 3). By CKD subtype, the analysis was based on 38 studies as mentioned above. HCV infection was associated with an 1.54-fold increased risk (OR, 95% CI, *P* : 1.54, 1.27-1.87, < 0.001) of having proteinuria in 7 cross-sectional studies, while no significance was observed in studies with eGFR of different cutoffs (*P* > 0.05). In longitudinal studies, a graded increased risk for incident CKD was noticed with reduced eGFR, that is, for eGFR of less than 60, 30 and 15 ml/min/1.73m^2^, the corresponding HR was 1.39 (95% CI, P: 1.14-1.69, 0.001) in 9 studies, 1.79 (95% CI, P: 0.91-3.51, 0.091) in 1 study and 2.30 (95% CI, P: 1.26-4.19, 0.007) in 5 studies, and there was no improvement in heterogeneity.

**Table 3:** Summary on stratified analyses according to country, CKD definition and median follow-up period respectively in cross-sectional and longitudinal studies

Subgroups	Cross-sectional studies			Longitudinal studies		
	Number of studies	OR, 95% CI, P	*I*^2^	Number of studies	HR, 95% CI, P	*I*^2^
***Country***						
East Asia	4	1.99, 1.23-3.20, 0.005	96.7%	4	1.69, 1.44-1.98, <0.001	54.2%
U.S.	11	1.04, 0.74-1.48, 0.811	99.2%	6	1.15, 1.02-1.31, 0.026	92.8%
Europe	0	NR	NR	2	1.89, 0.51-7.03, 0.345	99.0%
***CKD definition***						
Proteinuria	7	1.54, 1.27-1.87, <0.001	68.7%	0	NR	NR
eGFR <60 ml/min/1.73m2	12	1.11, 0.77-1.60, 0.567	99.4%	9	1.39, 1.14-1.69, 0.001	97.3%
eGFR <30 ml/min/1.73m2	3	1.08, 0.86-1.38, 0.502	67.1%	1	1.79, 0.91-3.51, 0.091	NR
eGFR <15 ml/min/1.73m2	1	1.22, 0.69-2.16, 0.500	NR	5	2.30, 1.26-4.19, 0.007	98.4%
***Median follow-up period***						
<5 years	NR	NR	NR	6	1.21, 1.03-1.43, 0.024	92.0%
≥5 years	NR	NR	NR	6	1.86, 1.19-2.89, 0.006	98.1%

Notes. CKD: chronic kidney disease; eGFR: estimated glomerular filtration rate; OR: odds ratio; HR: hazard ratio; 95% CI: 95% confidence interval; I2: inconsistency index; NR: not reported.

By country, the presence of HCV infection was associated with an 1.99-fold (95% CI, *P* : 1.23-3.20, 0.005) and 1.69-fold (95% CI, *P* : 1.44-1.98, < 0.001) increased risk of the prevalent (4 cross-sectional studies) and incident (4 longitudinal studies) CKD in East Asian countries (Taiwan and Japan), respectively (Table 3). Moreover in 6 U.S. longitudinal studies, the risk for incident CKD was marginally significant (HR, 95% CI, P: 1.15, 1.02-1.31, 0.026) and *I*^2^ was 92.8%. When the longitudinal studies were further grouped by median follow-up time at 5 years, the effect estimate was reinforced in long-term studies (HR, 95% CI, P: 1.86, 1.19-2.89, 0.006; *I*^2^ = 98.1%) relative to that in short-term studies (HR, 95% CI, P: 1.21, 1.03-1.43, 0.024; *I*^2^ = 92.0%).

### Meta-regression analyses

**Figure 2:**
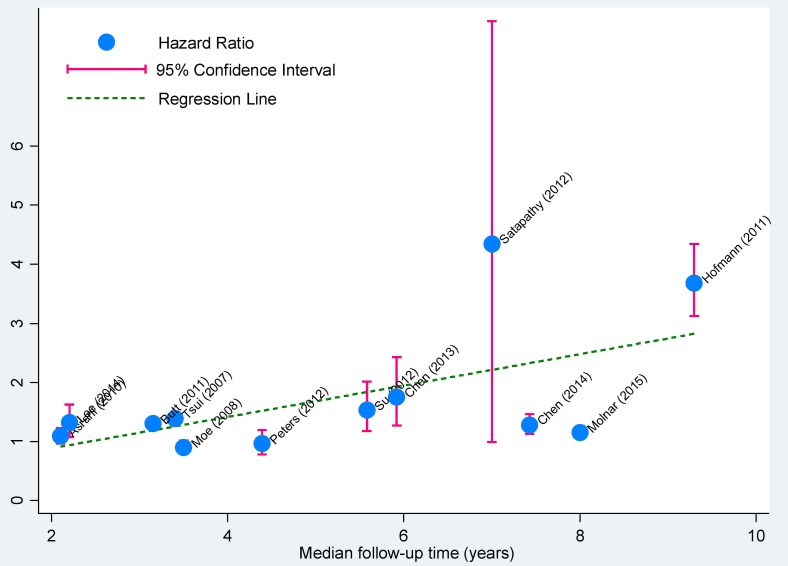
Correlation plot of median follow-up time with effect estimates in longitudinal studies

First, we one by one modeled all possible confounders including age, gender, diabetes mellitus, hypertension, country, CKD subtype and follow-up period (for longitudinal studies only), and interestingly found that the risk for incident CKD was significantly associated with the increased percentages of males (*P* = 0.020) and diabetes mellitus (*P* = 0.005), as well as the reduced eGFR (*P* = 0.017) and increased follow-up time (*P* = 0.002, Figure 2). We next modeled all possible confounders simultaneously and failed to detect any observable significance, which was likely attributed to the fact that meta-regression did not have the methodological rigor of a properly-designed study that was intended to test the effect of these covariates formally despite its capability to consider various covariates [31].

### Publication bias

**Figure 3:**
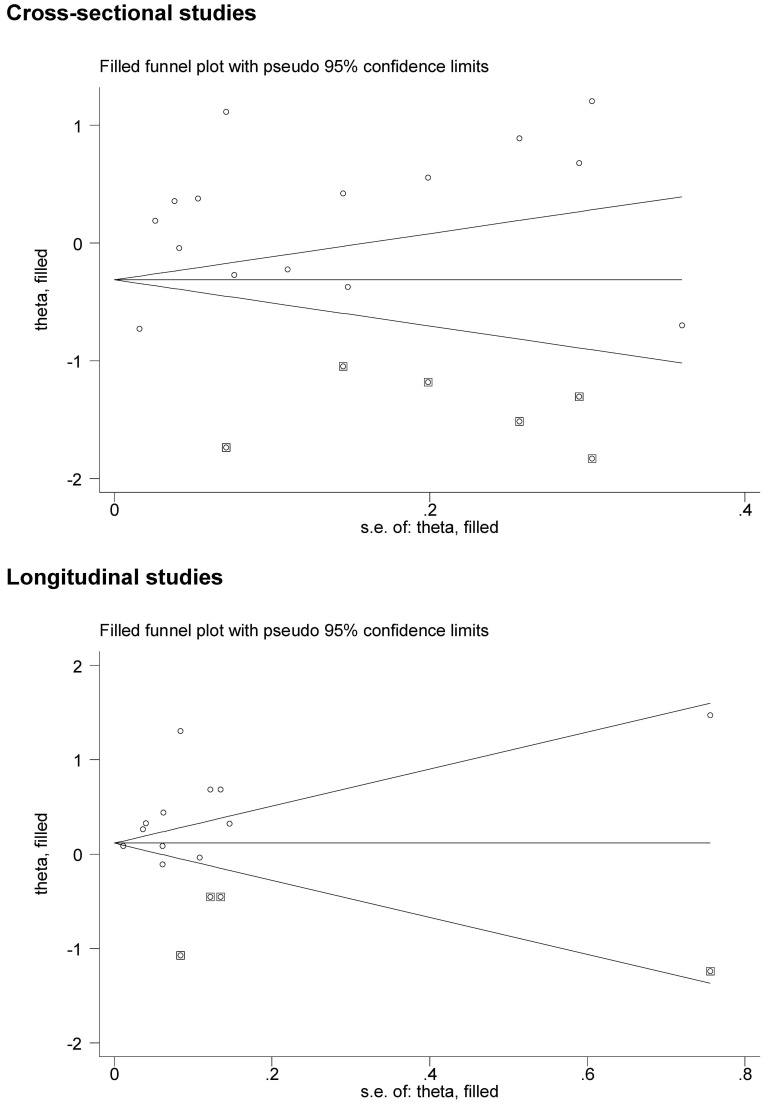
The filled funnel plots for the prevalence and incidence of CKD conferred by the presence of HCV infection Actual studies are denoted by the hollow circles, and potential missing studies in need to achieve symmetry are denoted by the solid squares. The theta in the y-axis represents the log(odds ratio) for cross-sectional studies and the log(hazard ratio) for longitudinal studies. The s.e. of theta in the x-axis represents the standard error of log(odds ratio or hazard ratio).

The filled funnel plots that reflected the evidence of selective publication are provided in Figure 3. The Egger's test suggested a marginally significant probability of publication bias for both longitudinal studies (*P* = 0.054) and cross-sectional (*P* = 0.064). As estimated, there were respectively 3 and 6 missing studies required to make the filled funnel plots symmetrical in longitudinal and cross-sectional studies. To account for the impact of these possible missing studies on the overall effect estimates, we employed the trim-and-fill analysis and identified a reduced risk conferred by HCV infection for prevalent CKD in simulated 21 cross-sectional studies (OR, 95% CI, P: 0.79, 0.59-1.06, 0.121), but an increased risk for incident CKD in simulated 16 longitudinal studies (HR, 95% CI, P: 1.16, 0.96-1.39, 0.118).

## DISCUSSION

The most noteworthy finding of this meta-analysis is the significant risk of experiencing incident CKD after HCV infection, with the lower eGFR and longer HCV exposure time entailing a greater risk. As far as we know, this is to-date the largest comprehensive met-analysis that has assessed the predisposition of HCV exposure to the occurrence and severity of CKD in the current literature.

As opposed to the significant effect estimate in cross-sectional studies in the latest meta-analysis by Park et al [11], we utilized a large sample size and failed to support the contributory role of HCV infection in the prevalence of CKD, in agreement with another recent more comprehensive meta-analysis by Fabrizi et al [12]. Although we and Fabrizi et al [12] both observed the independent predictive capability of HCV infection for prevalent proteinuria, we additionally identified a graded risk for incident CKD with the lower eGFR and longer HCV exposure time. This tendency is clinically plausible as HCV infection was associated with a wide range of extra-hepatic manifestations in various organs including the kidneys and it is highly prevalent among CKD patients under hemodialysis and in kidney transplantation recipients [32]. Also this finding lent some indirect credence for the likely detrimental impact of HCV infection in the development of CKD. In theory, several possible pathways have been proposed for the predisposition of HCV to extra-hepatic manifestations. Experimental data indicated that HCV can be conveyed by infected B-lymphocytes or exosomes to enter renal tissue for replication [33, 34] and cause kidney injury through cryoglobulins, HCV-antibody immune complexes, or amyloid deposition [5]. Moreover, many ingredients required for HCV attachment were found to be abundantly expressed in renal parenchyma [5]. Although the exact molecular mechanisms of how HCV infection entails the risk of CKD remain unclear, it is possible that systemic immune response to HCV infection might be one of the pathophysiological mechanisms.

A note of caution, however, should be made when interpreting our findings, because unexpectedly HCV exposure was nonsignificantly associated with the prevalence of CKD when analysis was restricted to cross-sectional studies. Some studies even observed that HCV infection was a protective factor for the prevalence of CKD [6, 7, 15, 16, 30]. By contrast in longitudinal studies, HCV-infected individuals had a 45% significantly increased risk of experiencing incident CKD after adjusting for traditional risk factors during 2 to 9 years of follow-up and even restricting analysis to multivariate-adjusted effect estimates. Actually, it is not uncommon in the literature to encounter such divergence between longitudinal and cross-sectional designs, even in the same study population. For example, HCV infection was associated with an increased risk of incident end stage renal disease (ESRD, eGFR < 15 ml/min/1.73m^2^), but a reduced risk of prevalent CKD (eGFR < 60 ml/min/1.73m^2^) in the study by Tsui et al [16]. A possible explanation for this case is that most sources of error due to confounding and bias are more common in cross-sectional studies than in longitudinal studies. For this reason, the findings from cross-sectional studies are often criticized. Moreover, such divergence between longitudinal and cross-sectional studies may also be proposed as a rational explanation for the negative correlation between HCV infection and CKD risk observed in the previous meta-analysis by Fabrizi et al [10].

Several possible limitations should be acknowledged for the interpretation of our meta-analytical findings. Firstly, we selected eligible studies from only English-language literature, and some well-designed studies published in the other languages might introduce a possible selection bias. Secondly, we cannot fully rule out all potential biases due to the unavailability of individual participant data in this study. Thirdly, although stratified and meta-regression analyses were conducted to explore and interpret diversity among the results of different studies, there was still no material improvement in heterogeneity. Fourthly, both filled funnel plots and Egger's tests indicated moderate evidence of publication bias; however taking into account the number and potential outcome of missing studies in the trim-and-fill analysis still produced significant effect sizes. Fifthly, it must be emphasized that nearly all eligible studies in this meta-analysis were conducted in the U.S. and Taiwan, thus the application of our findings cannot be extrapolated to populations in other continents.

In conclusion, this meta-analysis of 22 articles provides strong evidence for the significant risk of experiencing incident CKD after HCV infection, with the lower eGFR and longer HCV exposure time entailing a greater risk. It is of clinical importance to elucidate the molecular mechanisms underlying the HCV infection-CKD relationship, which will constitute an extremely promising field in life sciences. Importantly, treatment of HCV infection in CKD patients still remains a clinical challenge.

## MATERIALS AND METHODS

### Checklist

The conduct of this meta-analysis adheres to the guidelines listed in the Preferred Reporting Items for Systematic Reviews and Meta-Analyses (PRISMA) statement [35] (see the PRISMA checklist in Supporting Table S1).

### Search strategies

To ensure comprehensive literature coverage, we searched electronic databases including Medline (PubMed), EMBASE, Web of Science and Google-Scholar as of July 14, 2016 using the key terms ‘hepatitis C’, ‘HCV’, ‘anti-HCV positive status’, ‘chronic kidney disease’, ‘renal disease’, ‘CKD’, ‘renal/kidney impairment’, ‘renal/kidney insufficiency’, ‘renal/kidney failure’, ‘proteinuria’ and ‘microalbuminuria’. As a primary need, all potential articles gathered must be published in English language and performed in human beings. The reference lists of major original articles and reviews were manually checked to avoid potential missing hits.

### Inclusion criteria

The retrieved article was included if a cross-sectional or longitudinal study was designed to assess the incidence or prevalence of CKD or its graded stages in HCV-infected patients in comparison with controls who were not infected by HCV. The primary effect size was, if available, the ultimately-adjusted odds ratio (OR) or hazard ratio (HR) and the corresponding 95% confidence interval (95% CI), or was derived from a 2×2 contingency table with the counts of subjects with and without CKD under the presence or absence of HCV infection.

### Article selection

Based on pre-determined selection criteria, two authors (Min Li and Peiyuan Wang) independently identified eligible articles by reviewing the title or abstract of each retrieved article and if necessary the full text. If an article provided data on the basis of both cross-sectional and longitudinal scenarios or specific CKD stages, each was analyzed separately.

### Data abstraction

From each eligible article, two authors (Min Li and Peiyuan Wang) were in charge of abstracting pre-determined relevant information according to the results of within-group discussion and for the sake of accuracy this process was independently completed and checked for consistency. Any disagreement was settled with a consensus reached.

Abstracted data included first author's surname, year in publication, cohort or population name, follow-up time, the country where study subjects were enrolled, race/ethnicity, sample size, study design, the cutoff of eGFR to define CKD, adjusted effect estimate and its 95% CI, the counts of subjects with and without CKD under the presence or absence of HCV infection, age, gender, hypertension and diabetes mellitus if available.

### Statistical analyses

The effect-size estimate of each independent study was summarized in random-effects model that used the DerSimonian and Laird method [36]. The magnitude of between-study heterogeneity was represented by inconsistency index (I^2^) statistic, which is defined as the percentage of observed variability between studies that can be explained by heterogeneity rather than a chance finding. Stratified analyses by study design (cross-sectional studies and longitudinal studies), country (the U.S., East Asia and Europe) and median follow-up time (in longitudinal studies only: short-term: < 5 years and long-term: ≥ 5 years). Meta-regression analyses were further conducted to account for potential sources of clinical heterogeneity. The probability of publication bias was visually inspected by the filled funnel plots and statistically examined by the Egger's test at a significance level of 5%. The meta-analytical programs implemented in STATA software (StataCorp, TX, version 13.0) were employed for above statistical analyses. In addition, study power was estimated by the Power and Sample Size Calculations (PS) software (version 3.0) [37].

## SUPPLEMENTARY FIGURE AND TABLE




