# Supplementary material for: A systematic review and meta-analysis: Does hepatitis C virus infection predispose to the development of chronic kidney disease?
Source: Oncotarget. 2016 Oct 25;8(6):10692–702. doi: 10.18632/oncotarget.12896 (PMC5354692; doi:10.18632/oncotarget.12896)
Supplement: Supplementary file 1 [file oncotarget-08-10692-s001.pdf]

A systematic review and meta-analysis - Does hepatitis C virus infection predispose to the development of chronic kidney disease ?

Supplementary Material

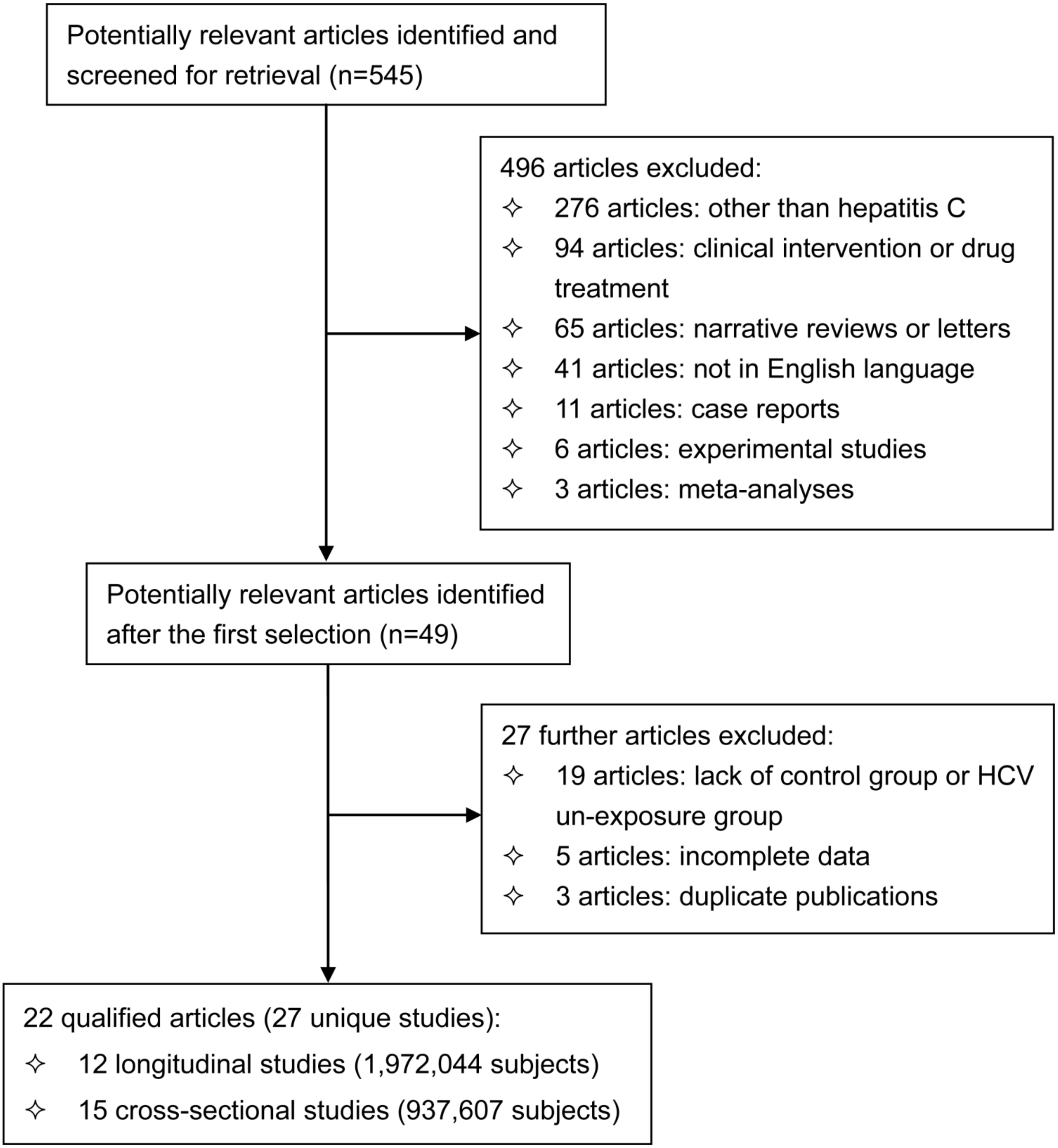

Supporting Figure S1. The PRISMA flow chart
